# Small breast epithelial mucin as a useful prognostic marker for breast cancer patients

**DOI:** 10.1515/biol-2022-0784

**Published:** 2023-11-23

**Authors:** Hui Hao, Lin Yang, Bingsheng Wang, Yinzhou Sang, Xueliang Liu

**Affiliations:** Department of Oncology, Cangzhou People’s Hospital, Cangzhou, 061000, China; Department of Pathology, Cangzhou People’s Hospital, Cangzhou, 061000, China; Breast Center, Cangzhou People’s Hospital, Cangzhou, 061000, China

**Keywords:** breast cancer, small breast epithelial mucin, immunohistochemistry, prognostic, biomarker

## Abstract

This study aimed to evaluate the clinical utility of small breast epithelial mucin (SBEM) as a prognostic biomarker in an independent patient cohort. The paraffin-embedded tissues and clinicopathological data of 105 patients with breast cancer were collected, and the expression of SBEM in breast cancer samples was detected by immunohistochemical staining. The correlations between clinicopathological variables and the expression of SBEM were analyzed, and its significance as a prognostic indicator for breast cancer patients was determined. Immunohistochemical staining revealed that SBEM was expressed mostly in the cytomembrane and cytoplasm, with markedly increased SBEM expression (≥4 points on staining intensity) observed in 34 of 105 breast cancer tissues (32.4%). Elevated expression of SBEM was found to be significantly associated with larger tumor size (*P* = 0.002), more frequent lymph node metastasis (*P* = 0.029), advanced tumor node metastasis stage (*P* = 0.005), reduced expression of the progesterone receptor (PR) (*P* = 0.002), and a higher Ki-67 index (*P* = 0.006). Survival analysis indicated that patients with elevated SBEM expression had worse overall survival (OS) (5-year OS rate: 50.5 vs 93.9% for high and low SBEM expression, respectively, *P* < 0.001) and disease-free survival (DFS) (5-year DFS rate: 52.8 vs 81.7% for high and low SBEM expression, respectively, *P* = 0.001) rates than those with low expression of SBEM. Univariate and multivariate Cox analyses demonstrated that elevated expression of SBEM (hazard ratio [HR] = 1.994, 95% confidence interval [CI]: 1.008–3.945, *P* = 0.047), tumor size (HR = 2.318, 95% CI: 1.071–5.017, *P* = 0.033), and PR status (HR = 0.195, 95% CI: 0.055–0.694, *P* = 0.012) were independent predictors of OS in breast cancer patients. Elevated expression of SBEM was associated with both aggressive tumor characteristics and poor survival, indicating its potential as a useful prognostic biomarker for breast cancer patients.

## 1 Introduction

Breast cancer is a common malignancy and the leading cause of cancer-related death in women. The disease is heterogeneous in terms of molecular features, morphology, and biological behavior [1,2,3]. Due to the lack of recognized symptoms and signs, breast cancer usually presents at an advanced stage, resulting in poor therapeutic efficacy and low survival outcomes [4,5,6]. Therefore, it is of great significance to identify novel prognostic biomarkers and therapeutic targets for breast cancer patients.

The mucin family are large proteins with heavy glycosylation, and it could be classified as membrane-bound (MUC1, MUC4, MUC13, and MUC16) and secretory types (MUC2, MUC5AC, MUC5B, and MUC6). The mucins form a chemical barrier in luminal surfaces of organs such as breast, pancreas, and gastrointestinal tract against infection and inflammation. As essential components of cells, mucin proteins also play important roles in cellular apoptosis, adhesion, and metastasis. It has been reported that mucins are used as specific diagnostic markers and therapeutic targets for human cancers. Small breast epithelial mucin (SBEM), also known as MUCL1, is a key member of the membrane-bound mucin family. SBEM is specifically expressed in salivary and mammary glands [7,8]. A previous study suggested that SBEM may be a valuable biomarker for bone marrow micrometastases in breast cancer patients [9,10]. Emerging evidence has also shown that SBEM levels are markedly increased in the peripheral blood of breast cancer patients in comparison with healthy controls [11]. By contrast, low levels of SBEM were more frequently found in breast cancer patients who underwent neoadjuvant chemotherapy [11]. In addition, SBEM was identified as an oncogene that promoted the migration and invasion of breast cancer cells by the regulation of the epithelial-to-mesenchymal transition [12]. However, the clinical value of SBEM as a prognostic biomarker has not yet been clarified. Therefore, the current study focused on the expression of SBEM in breast cancer tissues using immunohistochemistry and analyzed the correlations between its expression and clinicopathological characteristics as well as survival outcomes in breast cancer patients. Our findings may provide a valuable biomarker for the prognostic assessment and clinical treatment of breast cancer patients.

## 2 Materials and methods

### 2.1 Patients and clinical samples

A total of 105 patients who had been diagnosed and treated with surgical resection for breast cancer in our hospital between January 2016 and December 2018 were consecutively enrolled. All cases were pathologically diagnosed with invasive breast carcinoma, and the patients were eligible if they had no evidence of distant metastasis at the initial diagnosis. None of the patients received neoadjuvant radiotherapy, chemotherapy, or chemoradiotherapy before surgery. Patients with comorbid malignancies or incomplete clinicopathological and follow-up data were excluded. This study was approved by the Ethics Committee of our institution (No. K2020-003), and informed consent was obtained from all participants before surgery.

Paraffin-embedded tissues and clinicopathological data of 105 breast cancer patients were retrospectively collected. The demographic and clinicopathological features, including patient age, histological grade, tumor size, lymph node metastasis, anatomical tumor node metastasis (TNM) stage, expression of the estrogen receptor (ER) and progesterone receptor (PR), amplification status of human epidermal growth factor receptor 2 (HER2), Ki-67 expression, P53 mutation, and molecular subtype, were analyzed. The expression of ER, PR, and Ki-67 was detected by immunohistochemistry. The threshold of ER and PR positivity was defined as >10%, and cases showing nuclear staining of ≥14% of Ki-67 were classified as having high Ki-67 expression.


**Informed consent:** Informed consent has been obtained from all individuals included in this study.
**Ethical approval:** The research related to human use has been complied with all the relevant national regulations, institutional policies, and in accordance with the tenets of the Helsinki Declaration, and has been approved by the Ethics Committee of Cangzhou People’s Hospital (No. K2020-003).

### 2.2 Immunohistochemical analysis

Immunohistochemical staining for SBEM expression was performed using the streptavidin–peroxidase method according to both previous reports and manufacturers’ instructions.

Briefly, fixed tissue samples were embedded in paraffin and cut into 4 µm sections. The sections were then deparaffinized with a xylene solution and rehydrated in descending concentrations of ethanol. Antigen repair and retrieval were conducted with 0.01 M sodium citrate buffer (pH = 6.0) under heating for 30 min at 95°C in a microwave, after which the slides were blocked with 0.3% H_2_O_2_ solution for 10 min to inhibit the activity of endogenous peroxidase. After washing with phosphate-buffered saline, the sections were incubated with the primary antibody, polyclonal rabbit anti-SBEM (1:200, ab122530, Abcam, Cambridge, UK), overnight at 4°C. On the following day, the slides were incubated with the secondary antibody (anti-rabbit IgG) for 60 min at room temperature. Immunohistochemical responses were visualized by incubation with diaminobenzidine solution, and the sections were counterstained with hematoxylin. Finally, the slides were dehydrated in an alcohol gradient and fixed with xylene, followed by evaluation and imaging under a microscope.

The expression level of SBEM in breast cancer tissues was semi-quantitatively evaluated according to the intensity of immunohistochemical staining and the percentage of SBEM-positive cells. The staining intensity of SBEM was classified into four grades, namely, negative (= 0 points), weak (= 1 point), moderate (= 2 points), and strong (= 3 points). Five different visual fields were randomly selected, and 200 cells in each were counted. The staining proportions were scored as <5% (= 0 points), 5‒25% (= 1 point), 26‒50% (= 2 points), 51‒75% (= 3 points), and >75% (= 4 points). The immunohistochemical score for SBEM expression = staining intensity * the proportion of positive cells. <4 points was defined as negative SBEM expression, and ≥4 points was defined as positive expression of SBEM.

### 2.3 Statistical analysis

The patients were divided into negative- and positive-expression groups based on the SBEM immunohistochemical results. Correlations between clinicopathological variables and SBEM expression were summarized using a contingency table and analyzed by Pearson’s chi-squared or Fisher’s exact test. The primary outcomes for the survival analysis were overall survival (OS) and disease-free survival (DFS). The former was defined as the time from the date of surgical treatment to the date of death due to any cause or the last follow-up, while the latter was defined as the time interval from the date of surgical treatment to the date of the first postoperative tumor recurrence or metastasis. Survival differences between patients with negative and positive SBEM expression were evaluated by the Kaplan–Meier method with log-rank tests. In addition, univariate and multivariate Cox regression analyses were performed to identify the significant predictors of OS and DFS for breast cancer patients, and the data were expressed as the hazard ratio (HR) and 95% confidence interval (CI). Data processing and statistical analysis were performed using SPSS implemented with the SPSS version 23.0 (IBM Corp, Armonk, NY, USA). Two-sided *t*-tests were used for all statistical analyses, and a *P*-value of <0.05 was regarded as statistically significant.

## 3 Results

### 3.1 Expression of SBEM in breast cancer samples and its correlations with clinicopathological characteristics

**Figure 1:**
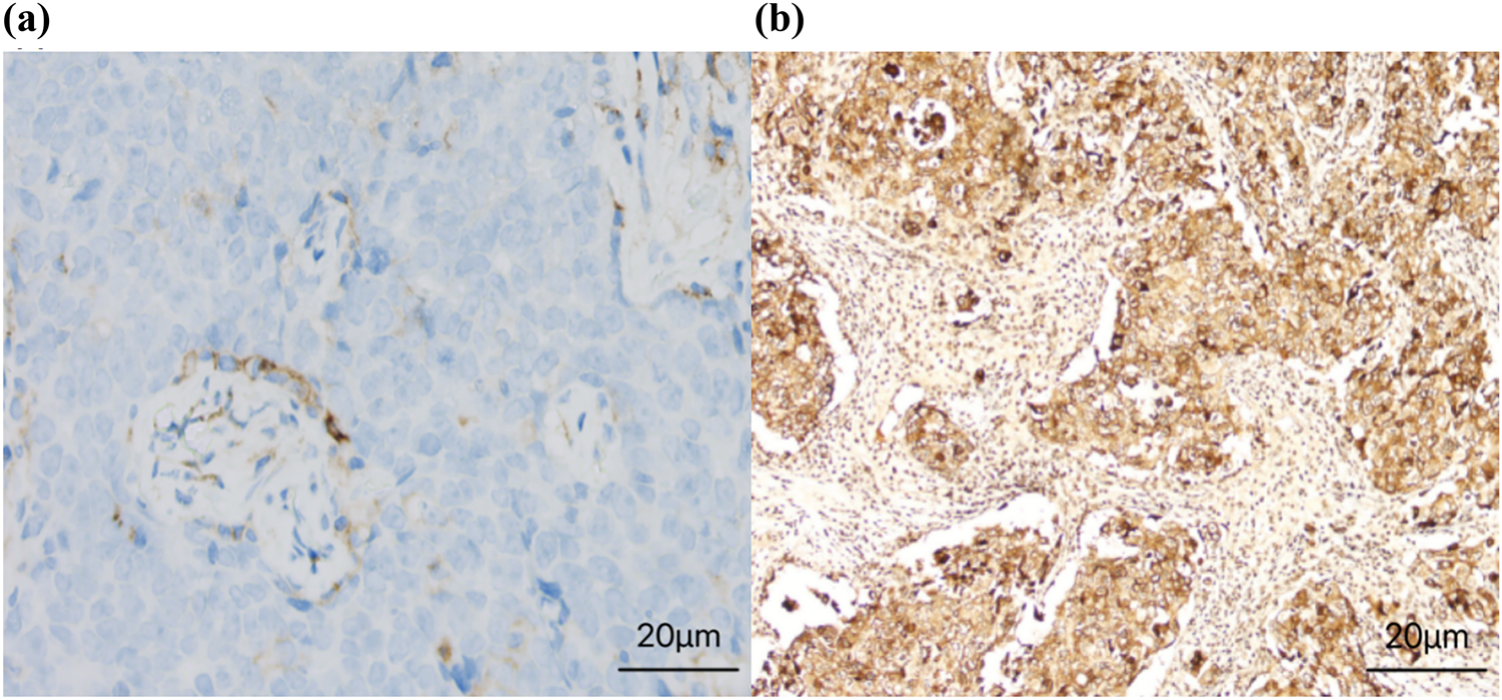
Representative images of SBEM expression in breast cancer tissues: (a) the negative expression of SBEM (×400) and (b) the positive expression of SBEM in immunohistochemical staining (×400).

Immunohistochemical staining showed that SBEM was mainly expressed in the cytomembrane and cytoplasm. Representative images of positive and negative SBEM expression in breast cancer samples are shown in Figure 1. Positive expression of SBEM was detected in 34 of 105 breast cancer tissues (32.4%).

The correlations between clinicopathological features and SBEM expression are summarized in Table 1. It is found that the positive expression of SBEM was significantly associated with larger tumor size (*P* = 0.002), frequent lymph node metastasis (*P* = 0.029), advanced TNM stage (*P* = 0.005), negative expression of PR (*P* = 0.002), and higher Ki-67 index (*P* = 0.006). However, there were no significant correlations between positive SBEM expression and other clinicopathological parameters such as age, ER status, histological grade, and molecular subtype (*P* > 0.05).

**Table 1:** Correlations between clinicopathological variables and the expression of SBEM in breast cancer

Variables	Total (*N* = 105)	SBEM expression		*χ* ^2^ value	*P* value
		Positive (*N* = 34)	Negative (*N* = 71)		
**Age (years)**				0.421	0.517
<50	29	8 (23.5%)	21 (29.6%)		
≥50	76	26 (76.5%)	50 (70.4%)		
**Tumor size (cm)**				9.385	**0.002**
≤2	44	7 (20.0%)	37 (54.7%)		
>2	61	27 (80.0%)	34 (45.3%)		
**Lymph node metastasis**				4.792	**0.029**
No	47	10 (29.4%)	37 (52.1%)		
Yes	58	24 (70.6%)	34 (47.9%)		
**TNM stage**				—	**0.005**
I	21	2 (6.7%)	19 (30.2%)		
II	71	24 (70.0%)	47 (69.8%)		
III	13	8 (23.3%)	5 (0.0%)		
**Histological grade**				—	0.885
G1	11	3 (8.8%)	8 (11.3%)		
G2	78	25 (73.5%)	53 (74.3%)		
G3	16	6 (17.6%)	10 (15.2%)		
**ER status**				3.509	0.061
Negative	42	18 (52.9%)	24 (33.8%)		
Positive	63	16 (47.1%)	47 (66.2%)		
**PR status**				9.832	**0.002**
Negative	54	25 (73.5%)	29 (40.8%)		
Positive	51	9 (26.5%)	42 (59.2%)		
**Molecular subtype**				—	0.247
HER2 (+)	52	17 (50.0%)	35 (49.3%)		
Triple-negative	31	13 (38.2%)	18 (25.4%)		
Luminal A	11	1(2.9%)	9(14.1%)		
Luminal B	11	3(8.8%)	7(11.3%)		
**Ki67 (%)**				7.510	**0.006**
<14%	27	3 (6.7%)	24 (26.4%)		
≥14%	78	31 (93.9%)	47 (73.6%)		
**P53**				0.626	0.429
Wild	75	26(76.5%)	49 (69.0%)		
Mutant	30	8 (23.5%)	22 (31.0%)		
**Relapse**				9.505	**0.002**
No	76	18 (52.9%)	58 (81.7%)		
Yes	29	16 (47.1%)	13 (18.3%)		
**Death**				11.249	**0.001**
No	91	24 (70.6%)	67 (94.4%)		
Yes	14	10 (29.4%)	4 (5.6%)		

*P* < 0.05, 0.01 or 0.001.

### 3.2 Expression of SBEM and survival outcomes in breast cancer patients

**Figure 2:**
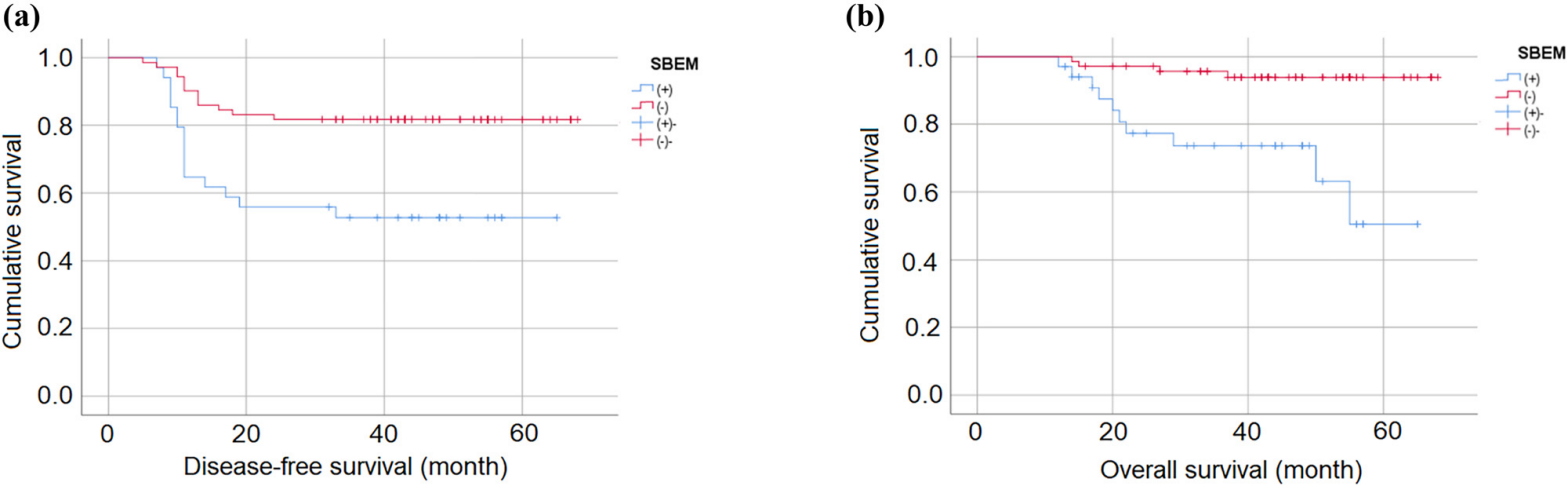
Kaplan–Meier curves showed the associations between SBEM expression and survival outcomes of breast cancer patients: (a) for DFS and (b) for OS.

The associations between SBEM expression and survival outcomes in breast cancer patients are shown in Figure 2. The Kaplan–Meier curves indicated that patients with positive expression of SBEM had worse OS and DFS than those with negative expression of SBEM. The 5-year OS rates of patients with positive and negative expression of SBEM were 50.5 and 93.9%, respectively (*P* < 0.001). The 5-year DFS rates of patients with positive and negative expression of SBEM were 52.8 and 81.7%, respectively (*P* = 0.001).

Univariate and multivariate Cox regression analyses for OS showed that positive expression of SBEM (HR = 1.994, 95% CI: 1.008–3.945, *P* = 0.047), tumor size (HR = 2.318, 95% CI: 1.071–5.017, *P* = 0.033), and PR status (HR = 0.195, 95% CI: 0.055–0.694, *P* = 0.012) were independent prognostic factors for breast cancer patients (Table 2). On the other hand, tumor size (HR = 2.232, 95% CI: 1.084–4.597, *P* = 0.029) and PR status (HR = 0.235, 95% CI: 0.076–0.730, *P* = 0.012) were identified as independent predictors of DFS for breast cancer patients. The multivariate analysis did not show an association between positive SBEM expression and DFS, with a HR of 1.773 (95% CI: 0.946–3.322, *P* = 0.074) (Table 3).

**Table 2:** Univariate and multivariate Cox analyses for predictors of OS in breast cancer patients

Variables	Univariate Cox analysis		Multivariate Cox analysis	
	HR (95% CI)	*P* value	HR (95% CI)	*P* value
**Age (years)**		0.374		
<50	Ref			
≥50	1.399 (0.668–2.933)			
**Tumor size (cm)**		**0.002**		**0.033**
≤2	Ref		Ref	
>2	2.951 (1.471–5.919)		2.318 (1.071–5.017)	
**Lymph node metastasis**		0.194		
No	Ref			
Yes	1.535 (0.804–2.931)			
**Histological grade**		0.266		
G1/G2	Ref			
G3	1.637 (0.687–3.902)			
**ER status**		0.436		
Negative	Ref			
Positive	0.783 (0.423–1.448)			
**PR status**		**0.039**		**0.012**
Negative	Ref		Ref	
Positive	0.491 (0.250–0.965)		0.195 (0.055–0.694)	
**Triple-negative breast cancer**		0.746		
No	Ref			
Yes	1.109 (0.592–2.081)			
**Ki67 (%)**		0.948		
<14%	Ref			
≥14%	0.974 (0.449–0.449)			
**P53**		0.601		
Wild	Ref			
Mutant	0.827 (0.405–1.687)			
**SBEM Expression**		**0.001**		**0.047**
Negative	Ref		Ref	
Positive	2.865 (1.546–5.311)		1.994 (1.008–3.945)	

*P* < 0.05, 0.01 or 0.001.

**Table 3:** Univariate and multivariate Cox analyses for predictors of DFS in breast cancer patients

Variables	Univariate Cox analysis		Multivariate Cox analysis	
	HR (95% CI)	*P* value	HR (95% CI)	*P* value
**Age (years)**		0.756		
<50	Ref			
≥50	1.110 (0.576–2.138)			
**Tumor size (cm)**		**0.002**		**0.029**
≤2	Ref		Ref	
>2	2.831 (1.463–5.497)		2.232 (1.084–4.597)	
**Lymph node metastasis**		0.524		
No	Ref			
Yes	1.209 (0.675–2.165)			
**Histological grade**		0.102		
G1/G2	Ref			
G3	1.891 (0.880–4.064)			
**ER status**		0.750		
Negative	Ref			
Positive	0.911 (0.514–1.616)			
**PR status**		0.079		**0.012**
Negative	Ref		Ref	
Positive	0.570 (0.304–1.068)		0.235 (0.076–0.730)	
**Triple-negative breast cancer**		0.716		
No	Ref			
Yes	0.895 (0.494–1.624)			
**Ki67 (%)**		0.951		
<14%	Ref			
≥14%	1.024 (0.479–2.193)			
**P53**		0.758		
Wild	Ref			
Mutant	0.902 (0.468–1.738)			
**SBEM expression**		**0.004**		0.074
Negative	Ref		Ref	
Positive	2.314 (1.299–4.124)		1.773 (0.946–3.322)	

*P* < 0.05, 0.01 or 0.001.

## 4 Discussion

The identification of specific biomarkers is useful for the early diagnosis and clinical management of breast cancer. It has been reported that SBEM is a novel tissue-specific protein in mammary glands, and the expression of SBEM is elevated in human breast cancer [8]. Current evidence indicates that SBEM is usually overexpressed in breast cancer tissues, cell lines, and peripheral blood samples from breast cancer patients [13,14]. Zhang et al. found that both mRNA and protein expression of SBEM was significantly increased in breast cancer tissues [15]. Several studies have reported a significant correlation between high SBEM expression and aggressive clinical features such as tumor size, lymph node metastasis, and clinical stage [11,15]. In a previous study, Liu et al. identified SBEM as a useful biomarker for predicting lymph node metastasis and micrometastasis in breast cancer patients [11]. This suggests that SBEM may provide valuable information for the prognostic assessment of breast cancer. However, little is known about the prognostic value of SBEM in breast cancer patients.

In this study, the protein expression of SBEM in breast cancer tissues was evaluated and the relationships between its expression and clinicopathological characteristics as well as survival outcomes of breast cancer patients were analyzed in an independent patient cohort. The data showed the positive expression of SBEM in 32.4% of samples, and its positive expression was significantly associated with larger tumor size, frequent lymph node metastasis, advanced TNM stage, negative expression of PR, and a higher Ki-67 index. Survival analysis revealed that the positive expression of SBEM was correlated with worse OS and DFS, suggesting its potential as a useful prognostic biomarker for breast cancer patients. These findings are consistent with those of Liu et al., who investigated the expression of SBEM in 87 triple-negative breast cancer (TNBC) tissues using immunohistochemistry, where they observed positive expression of SBEM in 58% of patients and found that it was an independent prognostic marker of OS and DFS in TNBC patients [16]. Moreover, high expression of SBEM was also detected in peripheral blood samples of breast cancer patients compared with healthy controls and other malignancies [11]. In view of its high specificity in breast tissue, evaluation of SBEM levels may thus be helpful for the early diagnosis, risk stratification, and therapeutic management of breast cancer patients.

As a potential oncogene, SBEM plays a critical role in breast cancer metastasis. Functional *in vitro* experiments demonstrated that the overexpression of SBEM markedly increased both the migration and invasion of MDA-MB-231 and MCF-7 cells [12]. Additionally, the levels of the epithelial marker E-cadherin were reduced, while those of mesenchymal markers such as vimentin, N-cadherin, and Twist were increased by the overexpression of SBEM [12]. These findings suggested that SBEM might promote breast cancer metastasis via the epithelial–mesenchymal transition. However, the biological functions and potential mechanisms of SBEM in the oncogenesis and progression of breast cancer need to be further explored in future research.

## 5 Conclusions

In conclusion, our findings suggested that the positive expression of SBEM was associated with both aggressive tumor characteristics and poor survival and that SBEM might thus be a valuable prognostic biomarker for breast cancer patients. As a tissue-specific protein, it is necessary to further evaluate the clinical utility of SBEM in larger patient cohorts.
